# Growth trajectories in monochorionic and dichorionic twins with weight discordance: a cohort study

**DOI:** 10.1007/s00431-025-06155-z

**Published:** 2025-05-01

**Authors:** Mariana Cortez Ferreira, Inês Marques Mariano, Andreia Marinhas, Isabel Santos Silva, Adelaide Taborda

**Affiliations:** 1 Neonatology Department, Maternidade Bissaya Barreto, Unidade Local de Saúde de Coimbra, Coimbra, Portugal; 2 Obstetrics Department, Maternidade Bissaya Barreto, Unidade Local de Saúde de Coimbra, Coimbra, Portugal

**Keywords:** Twin weight discordancy, Chorionicity, Perinatal outcomes, Neonatal outcomes, Children growth

## Abstract

Weight discordance is a known risk factor for poor perinatal and neonatal outcomes. However, its impact on growth patterns during childhood remains unclear. This study aimed to examine how weight discordance influences the growth of weight, length, and head circumference during the first 36 months of corrected age (CA), according to chorionicity. A retrospective cohort study of monochorionic and dichorionic twin pairs with weight discordance, born between January 1, 2012, and December 31, 2021, in a tertiary maternity hospital. Weight discordance was considered if there was a difference of more than 20% in estimated fetal weight or birth weight between the twins. *z*-scores for weight, length, and head circumference at birth and at 1, 6, 12, 18, 24, and 36 months of CA were retrieved, and a longitudinal comparison was performed between monochorionic and dichorionic twin pairs. Two hundred thirty-two infants were included: 64 monochorionic and 168 dichorionic. In monochorionic twins, the differences in weight, length, and head circumference between the smaller and larger co-twins closed six months earlier than in dichorionic twins. Head circumference *z*-scores became similar six months earlier than length *z*-scores, and length *z*-scores became similar six months earlier than weight *z*-scores. Overall, both small and large monochorionic and dichorionic twins showed increases in all growth parameters during the first 36 months CA, with a more notable increase in smaller monochorionic twins.

*Conclusion*: Inter-twin weight discordance was observed throughout the growth patterns of both larger and smaller twins. Smaller discordant twins showed adequate catch-up growth, leading to no differences in growth parameters compared to their larger co-twins by 36 months. This study highlights the role of chorionicity in the timing of resolution of growth disparities, with differences among dichorionic twins persisting longer.

**Table:** 

**What Is Known:** *• Weight discordance is a known risk factor for poor perinatal and neonatal outcomes, but its effect on childhood growth patterns remains unclear.* *• Previous studies showed persistent weight and length discrepancies during early childhood, but there is limited data on head circumference and time of growth discrepancy resolution.* **What Is New:** *• Head circumference z-scores in smaller twins caught up more quickly than length and weight z-scores, suggesting different rates of catch-up growth in different growth parameters.* *• Growth differences between dichorionic discordant twins persist longer than in monochorionic twins, but by 36 months, no differences remained in any growth parameter.*

**What Is Known:**

*• Weight discordance is a known risk factor for poor perinatal and neonatal outcomes, but its effect on childhood growth patterns remains unclear.*

*• Previous studies showed persistent weight and length discrepancies during early childhood, but there is limited data on head circumference and time of growth discrepancy resolution.*

**What Is New:**

*• Head circumference z-scores in smaller twins caught up more quickly than length and weight z-scores, suggesting different rates of catch-up growth in different growth parameters.*

*• Growth differences between dichorionic discordant twins persist longer than in monochorionic twins, but by 36 months, no differences remained in any growth parameter.*

## Introduction

The incidence of multiple pregnancies is steadily increasing, largely due to the rising age of mothers and the expanded use of assisted reproductive technologies (ART) [1–4]. Twin pregnancies carry higher maternal and neonatal risks, including growth abnormalities [1–3, 5–7].

Weight discordance is a significant difference in estimated fetal weight (EFW) or birth weight (BW) between twins. The optimal cut-off for defining discordance remains a topic of debate. However, the 20% cut-off proposed by the 2016 consensus of the American College of Obstetricians and Gynecologists (ACOG) and the International Society of Ultrasound in Obstetrics and Gynecology (ISUOG) is currently the most recognized definition [1–3].

The reported prevalence of discordant twins varies depending on the criteria used, but it is estimated to occur in 20–30% of all twin pregnancies [1–3, 8]. Previous studies have considered different weight discordance cut-offs varying between 10 and 25%, and most of them only considered postnatal BW discordance, ignoring prenatal EFW [6, 9, 10].

Inter-twin weight discordance has been recognized as an independent risk factor for complications during pregnancy, as well as in the perinatal and short-term neonatal periods [1–3, 5, 6]. Moreover, several studies have reported higher rates of fetal growth restriction (FGR) in discordant twins, which increases the risk of poor outcomes [3, 7, 9, 11].

Nevertheless, the association between weight discordance and long-term outcomes is less clear, and the data describing the growth patterns in discordant twins are limited. Previous studies showed significant heterogeneity regarding the criteria for defining weight discordance, as well as a frequent disregard for antenatal discordance in EFW and for growth parameters other than weight [6, 8, 9].

This study aims to analyze the effect of weight discordance on the growth patterns of weight, length, and head circumference in the larger and smaller twins during the first 36 months of corrected age (CA), according to chorionicity. As a secondary objective, it also aims to identify the pregnancy-related complications and perinatal and neonatal outcomes of monochorionic and dichorionic twins with weight discordance.

## Materials and methods

### Study design and patient selection

We conducted a retrospective cohort study of all consecutive twin infants with weight discordance born in a single tertiary maternity hospital from January 2012 to December 2021. The size and statistical power of the sample were calculated using the G*Power software application [12]. The following parameters were considered: *t*-tests; effect size: 0.5 (moderate effect—this magnitude was chosen due to the higher likelihood of clinical significance and based on prior studies where similar conditions yielded comparable effects on weight at 1 year) [1, 9]; α-level: 0.05; statistical power: 0.95; and two-tailed *p* value. Therefore, the initial size of the total sample was estimated at 42 monochorionic and 42 dichorionic twin infants.

The following cases were excluded: (1) twin pregnancies complicated by twin-to-twin transfusion syndrome (TTTS) or twin anemia polycythemia sequence, (2) multiple gestations of three or more fetuses, (3) neonates with major congenital malformations or chromosomal abnormalities related to morbidity and mortality, and (4) and children who died before 36 months of CA. If one twin met the exclusion criteria, their co-twin was automatically excluded from the study. Co-twins were paired with each other.

Clinical data were obtained through the review of the perinatal and neonatal medical records and through the follow-up assessment registered in the personal clinical file. These data are available in the electronic health record and were extracted directly from the online platform. Once discharged home, the Portuguese National Health System provides free well-child visits for all infants and children up to 18 years of age. These appointments include a complete medical examination and growth measurements (weight, length and head circumference) at standard time points (1 month, 2 months, 4 months, 6 months, 9 months, 12 months, 15 months, 18 months, 24 months, and 36 months), which are recorded in each child’s personal clinical record.

### Data collection

Prenatal and neonatal weight discordance was calculated using the formula: [(EFW or BW of the larger twin − EFW or BW of the smaller twin)/EFW or BW of the larger twin] × 100. The results were expressed as percentages, and values above 20% were considered weight discordance, according to the consensus of the ACOG and the ISUOG [13]. EFW was determined using ultrasound measurements of fetal biometry and was calculated using a modified Hadlock formula [14].

Chorionicity was determined between 11 + 0 and 13 + 6 weeks of gestation by ultrasound evaluation according to the number of placental masses or the presence of T-sign or lambda sign [13]. Gestational age was estimated using the first trimester ultrasound.

Sociodemographic characteristics (maternal age at delivery, parity, and preconception body mass index), use of ART (ovulation induction, in vitro fertilization, intracytoplasmic sperm injection, intrauterine insemination, egg donation, sperm donation, embryo donation, or frozen embryo transfer), and pregnancy-related complications (placenta previa, hypertension/preeclampsia, gestational diabetes, placental insufficiency, and chorioamnionitis) were assessed. Placental insufficiency was considered if any of the following markers were present on placental pathology examination: placental size <10 th percentile, decidual vasculopathy, fetal thrombotic vasculopathy, infarction, and/or distal villous hypoplasia [15].

Perinatal factors such as pregnancy surveillance, antenatal corticosteroid therapy, labor induction, prolonged premature rupture of membranes, oligohydramnios, FGR, cesarean delivery, sex, gestational age, appropriate for gestational age birth weight, five-minute Apgar score less than 7, and endotracheal intubation during neonatal resuscitation were also retrieved. Full pregnancy surveillance was considered if there were at least three antenatal sonographic assessments of fetal biometry. FGR was diagnosed according to the international Delphi consensus: onset before 32 weeks of gestation of an absent end-diastolic flow in the umbilical artery or a fetal abdominal circumference or estimated fetal weight below the 3rd centile or below the 10 th centile combined with abnormal Doppler findings in uterine or umbilical arteries [16].

Neonatal characteristics and morbidity were also explored. Admission to the neonatal intensive care unit (NICU), invasive or non-invasive respiratory support, and neonatal seizures were considered. Transient tachypnea of the newborn was defined as respiratory distress that began within the first 6 h of life, was transient and self-limited, and was resolved within the first postnatal week [17]. Neonatal respiratory distress syndrome was identified through chest X-ray findings consistent with the condition and corresponding arterial blood gas results [18]. Bronchopulmonary dysplasia was defined as oxygen need at 36 weeks postmenstrual age [19]. Neonatal sepsis was defined as clinical sepsis and abnormal laboratory findings (leukocyte count above 30,000/µL or under 5000/µL and C-reactive protein above 2 mg/dL), irrespective of blood culture results [20]. Patent ductus arteriosus was evaluated by an echocardiogram according to protocol or in case of clinical suspicion [21]. Hypoglycemia was diagnosed according to the American Academy of Pediatrics guidelines [22]. Necrotizing enterocolitis was classified according to the Modified *Bell’s* staging system and considered when the stage was ≥ IIA [23]. Retinopathy of prematurity (ROP) was graded using the International Classification of ROP and considered when a grade ≥ 3 was present [24]. Periventricular leukomalacia was classified according to De Vries et al. and considered when the grade was ≥ III [25]. Periventricular-intraventricular hemorrhage was graded using *Volpe*’s classification and considered when a grade III or a periventricular hemorrhagic infarction were present [26].

Weight, length, and head circumference at birth and at 1, 6, 12, 18, 24, and 36 months of CA were retrieved. Twin pairs were measured on the same day, by the same healthcare professional, and according to national growth measurement guidelines [27]. The *z*-scores of each measurement at birth for sex and gestational age were calculated based on Fenton 2013 growth charts [28]. The *z*-scores of each measurement for sex and CA were determined based on World Health Organization (WHO) 2006 growth standards [29].

### Data analysis

Statistical analysis was performed using IBM®SPSS® Statistics version 27. Categorical variables are presented as frequencies and percentages. Continuous variables are presented as means and standard deviations (SD) if normally distributed, while non-normally distributed data are presented as medians and interquartile ranges (IQR). Normal distribution was verified through the Kolmogorov–Smirnov test or skewness and kurtosis (maximum tolerated interval of − 1 to 1). Bivariate analysis was performed by using *χ*^2^ test (or Fisher exact test as appropriate) for categorical variables and t test for continuous variables, as all variables included in the analysis were normally distributed. The paired-samples *t*-test was used to analyze the growth patterns of the smaller and larger twin. This analysis takes into account that the observations between the co-twins are not independent.

All reported *p* values are two-tailed with values inferior to 0.05 indicating statistical significance.

### Ethical approval

Approval was obtained from the local Ethics Committee (process number 2024-ESI.SF-72). The institutional review board waived the requirement for informed consent due to the retrospective nature of the study.

## Results

**Fig. 1:**
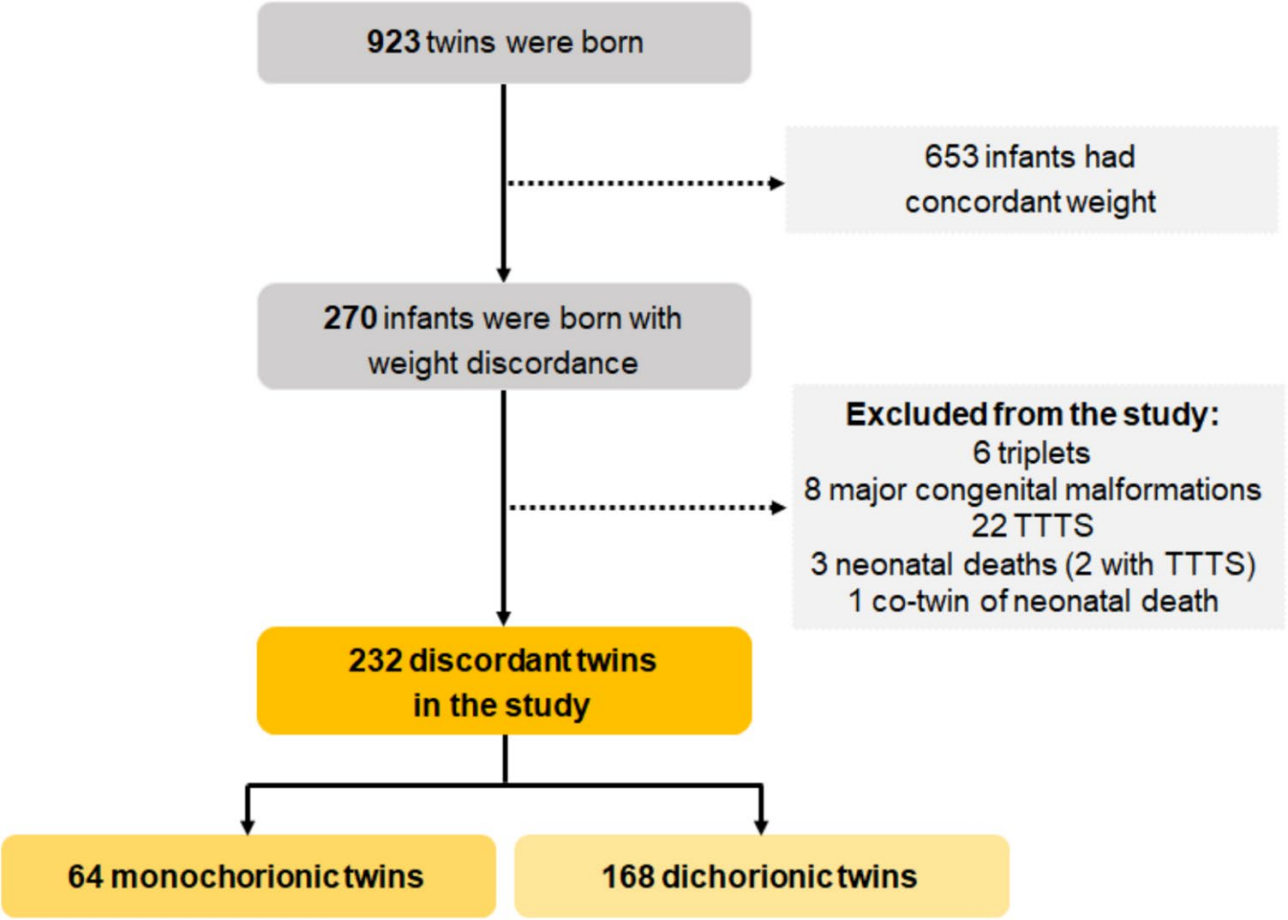
Flowchart of patient selection. TTTS, twin-to-twin transfusion syndrome

During the study period 923 twins were born, of which 270 (29.3%) were discordant. Thirty-eight discordant twins were excluded, 3 (1.1%) due to death before discharge. The final sample size consisted of 232 infants: 64 (27.6%) monochorionic and 168 (72.4%) dichorionic twins (Fig. 1).

### Pregnancy-related complications and perinatal and neonatal outcomes

The median weight discordance was 26% (IQR 14). ART was used in 1.7% of all pregnancies. FGR occurred in 44 (19.0%) cases, and 65.5% of all pregnancies underwent labor induction, with more than one-third (34.2%) induced due to weight concerns.

Ninety-four infants (40.5%) were born through emergency cesarean delivery, and 53.0% of all newborns were admitted to the NICU.

Preterm birth occurred in 170 (73.3%) cases: 34 (20.0%) were very preterm infants, and of those, 2 were extreme preterm infants.

There were no differences in perinatal and neonatal outcomes between monochorionic and dichorionic twins with weight discordance, despite the higher prevalence of FGR, NICU admission, transient tachypnea of the newborn, and the need for non-invasive respiratory support among monochorionic twin pairs. The remaining baseline characteristics and outcomes for the entire sample and separately for the smaller and larger twins are shown in Tables 1 and 2, respectively.

**Table 1:** Characteristics of the overall sample

	All infants (*n* = 232)	Monochorionic (*n* = 64)	Dichorionic (*n* = 168)	*p* value*
**Maternal characteristics and pregnancy-related complications**				
Maternal age—mean ± SD [years]	33.7 ± 6.0	33.3 ± 6.1	33.9 ± 6.0	0.558
Preconception BMI—mean ± SD [kg/m^2^]	25.9 ± 4.5	26.1 ± 5.0	25.9 ± 4.3	0.805
Nulliparous—*n* (%)	128 (55.2)	25 (39.1)	103 (61.3)	**0.002**
Placental insufficiency—*n* (%)	62 (26.7)	16 (25.0)	46 (27.4)	0.265
Fetal growth restriction—*n* (%)	44 (19.0)	18 (28.1)	26 (15.5)	**0.040**
Hypertension/preeclampsia—*n* (%)	46 (19.8)	11 (17.2)	35 (20.8)	0.534
Gestational diabetes—*n* (%)	27 (11.6)	5 (7.8)	22 (13.1)	0.262
Chorioamnionitis—*n* (%)	12 (5.3)	2 (3.1)	10 (6.0)	0.517
Placenta previa—*n* (%)	0 (0.0)	0 (0.0)	0 (0.0)	–
**Perinatal characteristics**				
Full pregnancy surveillance—*n* (%)	230 (99.1)	64 (100.0)	166 (98.8)	1.000
Antenatal corticosteroid therapy—*n* (%)	112 (48.3)	36 (56.3)	76 (45.2)	0.134
Threatened preterm labor—*n* (%)	66 (28.4)	18 (28.1)	48 (28.6)	0.946
Labor induction—*n* (%)	152 (65.5)	42 (65.6)	110 (65.5)	0.983
PPRM—*n* (%)	26 (11.2)	6 (9.4)	20 (11.9)	0.685
Oligohydramnios—*n* (%)	8 (3.4)	1 (1.6)	7 (4.2)	0.447
Cesarean delivery—*n* (%)	145 (62.5)	43 (67.2)	102 (60.7)	0.363
Male—*n* (%)	108 (46.6)	25 (39.1)	83 (49.4)	0.158
Gestational age at birth—mean ± SD [weeks]	33.1 ± 2.2	34.1 ± 2.7	34.8 ± 2.2	0.060
Birth weight—mean ± SD [g]	1635 ± 475	1775 ± 606	2060 ± 525	** < 0.001**
AGA birth weight—*n* (%)	144 (62.1)	38 (59.4)	106 (63.1)	0.602
Length at birth—mean ± SD [cm]	40.7 ± 3.8	41.5 ± 4.8	43.2 ± 3.4	**0.008**
HC at birth—mean ± SD [cm]	29.4 ± 2.3	30.0 ± 2.7	31.1 ± 2.3	**0.003**
Endotracheal intubation—*n* (%)	3 (1.3)	1 (1.6)	6 (3.6)	0.677
**Neonatal characteristics and morbidity**				
Admission to the NICU—*n* (%)	123 (53.0)	43 (67.2)	80 (47.6)	**0.008**
Invasive respiratory support—*n* (%)	18 (7.8)	8 (12.5)	10 (6.0)	0.105
Non-invasive respiratory support—*n* (%)	41 (17.7)	18 (28.1)	23 (13.7)	**0.010**
Transient tachypnea of the newborn—*n* (%)	40 (17.2)	18 (28.1)	22 (13.1)	**0.007**
Neonatal respiratory distress syndrome—*n* (%)	16 (6.9)	7 (10.9)	9 (5.4)	0.151
Bronchopulmonary dysplasia—*n* (%)	2 (0.9)	0 (0.0)	2 (1.2)	1.000
Sepsis—*n* (%)	6 (2.6)	1 (1.6)	5 (3.0)	1.000
Treated patent ductus arteriosus—*n* (%)	5 (2.2)	3 (4.7)	2 (1.2)	0.130
Treated hypoglycemia—*n* (%)	6 (2.6)	3 (4.7)	3 (1.8)	0.351
Necrotizing enterocolitis stage ≥ IIA—*n* (%)	2 (0.9)	0 (0.0)	2 (1.2)	1.000
Retinopathy of prematurity grade ≥ 3—*n* (%)	0 (0.0)	0 (0.0)	0 (0.0)	–
Periventricular leukomalacia grade ≥ III—*n* (%)	0 (0.0)	0 (0.0)	0 (0.0)	–
PIVH grade III or PVHI—*n* (%)	0 (0.0)	0 (0.0)	0 (0.0)	–
Seizures—*n* (%)	1 (0.4)	0 (0.0)	1 (0.6)	0.549

^*^p value comparing monochorionic and dichorionic infants

*Bold values indicate statistically significant difference (p < 0.05). AGA* appropriate for gestational age, *BMI* body mass index, *HC* head circumference, *IQR* interquartile range, *NICU* neonatal intensive care unit, *PIVH* periventricular-intraventricular hemorrhage, *PPRM* prolonged premature rupture of membranes, *PVHI* periventricular hemorrhagic infarction, *SD* standard deviation

**Table 2:** Baseline characteristics and neonatal morbidity of the smaller and larger twin in monochorionic and dichorionic dyads

	Monochorionic twins (*n* = 64)			Dichorionic twins (*n* = 168)		
	Small twin	Large twin	*p* value	Small twin	Large twin	*p* value
**Prenatal and perinatal characteristics**						
Fetal growth restriction—*n* (%)	16 (50.0)	2 (6.3)	** < 0.001**	19 (22.6)	6 (7.1)	**0.002**
Male—*n* (%)	12 (37.5)	12 (37.5)	1.000	25 (29.8)	57 (67.9)	** < 0.001**
Birth weight—mean ± SD [g]	1530 ± 525	2021 ± 589	** < 0.001**	1797 ± 444	2311 ± 468	** < 0.001**
AGA birth weight—*n* (%)	8 (25.0)	30 (93.8)	** < 0.001**	26 (31.0)	79 (94.0)	** < 0.001**
Length at birth—mean ± SD [cm]	39.8 ± 5.2	43.1 ± 3.7	**0.004**	41.9 ± 3.3	44.5 ± 2.9	** < 0.001**
HC at birth—mean ± SD [cm]	29.4 ± 2.7	30.7 ± 2.5	0.051	30.4 ± 2.3	31.7 ± 2.1	** < 0.001**
Endotracheal intubation—*n* (%)	1 (3.1)	0 (0.0)	0.492	3 (3.6)	2 (2.4)	0.871
**Neonatal characteristics and morbidity**						
Admission to the NICU—*n* (%)	23 (71.9)	20 (62.5)	0.424	41 (48.8)	38 (45.2)	0.328
Invasive respiratory support—*n* (%)	6 (18.8)	2 (6.3)	0.257	4 (4.8)	5 (6.0)	1.000
Non-invasive respiratory support—*n* (%)	7 (21.9)	11 (34.4)	0.266	7 (8.3)	15 (17.9)	0.105
Transient tachypnea of the newborn—*n* (%)	7 (21.9)	11 (34.4)	0.266	2 (2.4)	20 (23.8)	** < 0.001**
Neonatal respiratory distress syndrome—*n* (%)	4 (12.5)	3 (9.4)	1.000	4 (4.8)	4 (4.8)	1.000
Bronchopulmonary dysplasia—*n* (%)	0 (0.0)	0 (0.0)	-	2 (2.4)	0 (0.0)	0.428
Sepsis—*n* (%)	1 (3.1)	0 (0.0)	0.316	2 (2.4)	3 (3.6)	0.871
Treated patent ductus arteriosus—*n* (%)	0 (0.0)	3 (9.4)	0.238	1 (1.2)	1 (1.2)	1.000
Treated hypoglycemia—*n* (%)	3 (9.4)	0 (0.0)	0.238	2 (2.4)	1 (1.2)	0.607
Necrotizing enterocolitis stage ≥ IIA—*n* (%)	0 (0.0)	0 (0.0)	-	1 (1.2)	1 (1.2)	1.000
Seizures—*n* (%)	0 (0.0)	0 (0.0)	-	0 (0.0)	1 (1.2)	1.000

*Bold values indicate statistically significant difference (p < 0.05). AGA* appropriate for gestational age, *HC* head circumference, *NICU* neonatal intensive care unit, *SD* standard deviation

### Growth patterns during the first 36 months of corrected age

**Fig. 2:**
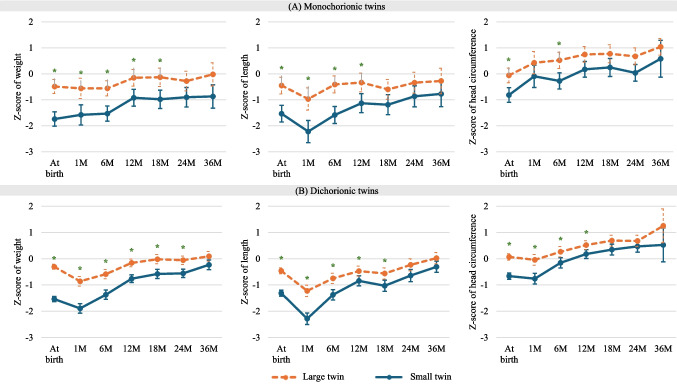
Growth patterns of small and large discordant monochorionic (**A**) and dichorionic (**B**) twins during the first 24 months of corrected age. * indicates a statistically significant difference (*p* < 0.05). M, months. Monochorionic twins showed persistent differences in weight, height, and head circumference z-scores up to 18, 12, and 6 months of corrected age, and dichorionic twins showed these differences up to 24, 18, and 12 months of corrected age, respectively

The mean *z*-scores and SD of weight, length, and head circumference at birth and at 1, 6, 12, 18, 24, and 36 months of CA are presented in Fig. 2.

At birth, the *z*-scores of weight, length, and head circumference were significantly higher in the large infants, regardless of chorionicity (Fig. 2). In monochorionic twins, significant differences in weight *z*-scores persisted until the 18 th month of CA, while height *z*-scores remained different among twin pairs until the 12 th month of CA, and head circumference *z*-score differences disappeared after the 6 th month of CA. On the other hand, in dichorionic twins, significant differences in weight *z*-scores among twin pairs persisted throughout the first 24 months of CA, while disparities in height *z*-scores continued until the 18 th month of CA, and differences in head circumference *z*-scores disappeared after the 12 th month of CA.

The *z*-scores for growth parameters (weight, length, and head circumference) increased in both small and large monochorionic and dichorionic twins over the first 36 months CA, although the difference remained significant for a longer period in the dichorionic twin pairs.

**Fig. 3:**
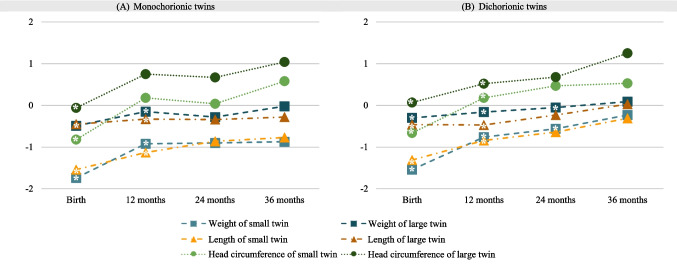
Growth patterns (*z*-scores) at birth and at 12, 24 and 36 months of corrected age among small and large discordant monochorionic (**A**) and dichorionic (**B**) twins. * indicates a statistically significant difference (*p* < 0.05). At birth, smaller and larger twins showed significant differences in all growth parameters; at 12 months of corrected age, only head circumference z-scores became irrelevant in the monochorionic twins; at 24 months of corrected age, only weight z-scores remained different in the dichorionic group; and at 36 months of corrected age, no growth parameter showed a significant difference between the smaller and larger twins

In monochorionic twins, the mean weight discordance decreased from 30.7% at birth to 12.2% at 36 months CA, the mean length discordance decreased from 9.2% at birth to 2.3%, and head circumference discordance decreased from 5.8% at birth to 1.1%. In dichorionic twins, the mean weight discordance decreased from 30.3% at birth to 11.0% at 36 months CA, the mean length discordance decreased from 6.8 to 2.9%, and head circumference discordance decreased from 4.8 to 2.5%. At 36 months CA, there was no significant difference between the twins, regardless of chorionicity, in any of the growth parameters. The total changes in growth parameters from birth to 36 months of corrected age are shown in Fig. 3.

The evolution of the mean *z*-scores for each growth parameter over time and the point at which the differences between the twins became insignificant are described in Table 3.

**Table 3:** Z-scores of weight, length, and head circumference of the small and large twins at birth and during the first 36 months of corrected age

	Monochorionic twins (*n* = 64)			Dichorionic twins (*n* = 168)		
z-score of weight—mean ± SD	Small twin	Large twin	*p* value	Small twin	Large twin	*p* value
At birth	− 1.74 ± 1.07	− 0.49 ± 1.15	** < 0.001**	− 1.54 ± 0.64	− 0.30 ± 0.63	** < 0.001**
At 1 month	− 1.58 ± 1.26	− 0.56 ± 1.59	** < 0.001**	− 1.89 ± 1.07	− 0.86 ± 1.19	** < 0.001**
At 6 months	− 1.53 ± 1.22	− 0.56 ± 0.93	** < 0.001**	− 1.37 ± 1.11	− 0.59 ± 0.97	** < 0.001**
At 12 months	− 0.92 ± 1.37	− 0.15 ± 1.12	** < 0.001**	− 0.76 ± 0.93	− 0.16 ± 0.82	** < 0.001**
At 18 months	− 0.98 ± 1.35	− 0.13 ± 1.13	** < 0.001**	− 0.58 ± 1.03	− 0.02 ± 0.81	**0.002**
At 24 months	− 0.90 ± 1.45	− 0.28 ± 1.14	0.055	− 0.56 ± 0.85	− 0.05 ± 0.87	**0.002**
At 36 months	− 0.87 ± 1.36	− 0.02 ± 1.14	0.060	− 0.23 ± 0.90	0.09 ± 0.90	0.083
Delta *z*-score from birth to 36 months	0.87	0.47	0.171	1.31	0.39	** < 0.001**
***z*-score of height**—**mean ± SD**						
At birth	− 1.54 ± 1.41	− 0.45 ± 1.16	** < 0.001**	− 1.31 ± 0.71	− 0.46 ± 0.73	** < 0.001**
At 1 month	− 2.22 ± 1.37	− 0.97 ± 1.71	** < 0.001**	− 2.28 ± 1.37	− 1.22 ± 1.33	** < 0.001**
At 6 months	− 1.59 ± 1.16	− 0.41 ± 1.22	** < 0.001**	− 1.37 ± 1.21	− 0.75 ± 1.02	**0.002**
At 12 months	− 1.13 ± 1.48	− 0.33 ± 1.31	**0.001**	− 0.84 ± 1.23	− 0.47 ± 1.04	**0.049**
At 18 months	− 1.19 ± 1.43	− 0.60 ± 1.30	0.054	− 1.03 ± 1.16	− 0.56 ± 1.09	**0.029**
At 24 months	− 0.86 ± 1.55	− 0.34 ± 1.23	0.060	− 0.64 ± 1.26	− 0.23 ± 1.14	0.080
At 36 months	− 0.77 ± 1.53	− 0.28 ± 1.17	0.072	− 0.31 ± 0.92	0.03 ± 1.11	0.123
Delta *z*-score from birth to 36 months	0.77	0.17	0.095	1.00	0.49	0.066
***z*-score of head circumference**—**mean ± SD**						
At birth	− 0.82 ± 0.86	− 0.06 ± 1.37	**0.004**	− 0.66 ± 0.73	0.07 ± 0.79	** < 0.001**
At 1 month	− 0.10 ± 1.43	0.43 ± 1.66	0.066	− 0.76 ± 1.24	− 0.04 ± 1.26	**0.001**
At 6 months	− 0.27 ± 1.11	0.52 ± 1.16	** < 0.001**	− 0.16 ± 1.15	0.27 ± 1.08	**0.034**
At 12 months	0.18 ± 1.14	0.75 ± 1.17	0.055	0.18 ± 0.95	0.52 ± 1.02	**0.042**
At 18 months	0.25 ± 1.23	0.77 ± 1.24	0.070	0.35 ± 1.06	0.69 ± 1.10	0.099
At 24 months	0.04 ± 1.11	0.67 ± 1.12	0.054	0.47 ± 0.95	0.68 ± 1.07	0.338
At 36 months	0.58 ± 1.58	1.04 ± 0.71	0.193	0.53 ± 1.33	1.25 ± 1.00	0.284
Delta z-score from birth to 36 months	1.40	1.10	0.739	1.19	1.18	0.715

*Bold values indicate statistically significant difference (p < 0.05). SD* standard deviation

## Discussion

### Main findings

Our findings suggest that (1) inter-twin weight discordance during pregnancy and at birth is associated with differences in the growth patterns among twin pairs during the first 36 months of CA, (2) its impact varies according to chorionicity, and (3) the differences in *z*-scores varied according to each growth measurement.

The growth patterns of weight, length, and head circumference differed significantly between the two co-twins, and chorionicity played an important role in the growth patterns. Previous studies have reported different pathogenic mechanisms underlying weight discordance according to chorionicity [1–3, 5, 6, 8, 30]. While in monochorionic twins, inequality in placental sharing and placental vascular anastomoses with hemodynamic imbalance are the main causes of inter-twin growth discordance, in dichorionic twins, different genetic backgrounds leading to constitutional inter-twin differences in growth potential and placental insufficiency are significant factors in the development of weight discordance [1–3, 6]. A prospective multicenter study by Kent et al. showed that while growth discordance in monochorionic twins is probably related to uneven distribution of placental mass between the two fetuses, unusual cord insertion sites, or abnormal vascular connections, in dichorionic twins, these growth differences are due to underlying uteroplacental insufficiency selectively affecting one of the twins [30].

Victoria et al. reported smaller total placental mass in severely discordant twins and greater morbidity in severely discordant monochorionic twins, with significantly higher rates of very preterm birth than in dichorionic twins [31].

In our study, monochorionic twins showed a narrowing gap in weight, length, and head circumference six months earlier than dichorionic twins. Since all monochorionic twins are monozygotic [32], their identical genetic background and similar constitutional growth potential may facilitate an earlier catch-up growth in the smaller twin.

Our findings are in line with those of some previous studies [1, 9, 10, 33]. Of note, Lim et al. [1] reported that differences in the *z*-scores of weight and height among co-twins became insignificant after discharge in monochorionic twins, while in dichorionic twins these differences were still found at 24 months of CA. However, this study did not take into account the antenatal discordance in EFW, did not evaluate head circumference, and did not conduct a follow-up longer than 24 months to determine when the discrepancy in dichorionic twins became irrelevant. Mazkereth et al. [9] conducted a retrospective study of discordant preterm twins and showed a rapid catch-up of the smaller twin in all growth parameters, with the gaps between the smaller twins and their larger co-twins being significantly smaller by 1 year of age. Similarly, a prospective study conducted by Ross et al. [10] showed that the smaller discordant twins experienced appropriate catch-up growth during the first 3 years of life. Nevertheless, both studies used a different definition of growth discordance. As this definition has been extensively debated, the criteria in previous studies vary. We believe that by using the definition proposed by the ACOG and the ISUOG consensus, we were able to more accurately evaluate the prognostic value of antenatal size discordance [6, 13].

In addition, regardless of chorionicity, the differences in *z*-scores varied according to each measurement: the differences in head circumference *z*-scores became insignificant between the twin pairs six months earlier than the differences in length *z*-scores, and the differences in length *z*-scores became similar six months earlier than the differences in weight *z*-scores. In fact, the catch-up in head circumference reached a *z*-score of nearly 0 at 6 months of CA in all twin pairs. This finding is of major significance, as previous studies have shown an association between larger head circumferences and higher cognitive function, academic performance, and improvements in educational attainment and employment [34–36].

Finally, we found that *z*-scores for all growth parameters (weight, length, and head circumference) increased in both small and large monochorionic and dichorionic twins during the first 36 months of CA, with a more prominent increase in the small monochorionic twins. Much of the available data on growth patterns among discordant twins comes from studies that only evaluated weight and length, with limited data available regarding head circumference growth. Groene et al. [33] conducted a longitudinal cohort study of discordant monochorionic twins with FGR and reported rapid catch-up growth in head circumference and body mass index in the first year of life, whereas rapid catch-up growth in height was prolonged into the second year of life. In this study, the authors concluded that despite modest persistent differences in all growth parameters, both smaller and larger twins reach their target height range by adolescence. On the other hand, other authors did not find differences regarding the different growth parameters [1, 8, 10]. Nevertheless, these papers significantly differ from our study regarding the growth discordance definition, the patient selection, the assessment ages, and the follow-up assessment tools. Hence, the comparison between our results and the findings of these other papers must be interpreted with caution.

These findings are clinically relevant and may have potential implications for pediatric follow-up protocols of weight discordant twins. Although the catch-up growth of the smaller twin may take longer than previously reported in some studies (especially in dichorionic twins), it is expected in the majority of cases. This data may provide additional reassurance to both parents and healthcare professionals regarding the growth trajectories of discordant twins. Nevertheless, while a watchful waiting approach appears to be a reasonable strategy in early childhood, further research is needed to confirm its long-term safety.

### Other findings

FGR was found in 28% of the monochorionic twins and 15% of the dichorionic twins and was the only significant prenatal difference between groups (*p* = 0.040). Previous studies have reported higher rates of FGR in monochorionic and dichorionic discordant twins, ranging from 40 to 55% [2, 3]. Although higher rates were reported in the monochorionic group, no statistical difference was found. Nevertheless, the FGR definition was not reported, and both studies were based on the Korean population.

### Strengths and limitations

To the best of our knowledge, this is one of the few studies that aims to determine the effect of inter-twin weight discordance on the growth patterns of large and small twins during early childhood using the criteria defined by the ACOG and the ISUOG consensus [13]. Therefore, by using antenatal discordance in EFW as proposed by the consensus, rather than relying solely on birth weight discrepancy as most available studies have done, we believe we have more accurately estimated the prognostic value of inter-twin weight discordance. In addition, we conducted a comprehensive study of all growth parameters (weight, length, and head circumference) from birth until the discrepancy between the twin pairs became negligible using the latest updated guidelines on growth charts recommended by the Portuguese Neonatal Society [37]. Additionally, we used *z*-scores, which offer a standardized method for assessing and comparing growth across different populations, age groups, and genders [29].

This study also has limitations. The study was conducted in a single tertiary maternity, and as such, these findings may be skewed and must be interpreted with caution. As a retrospective study, diagnoses and comorbidities may have been underreported. However, since the data regarding growth during early childhood was available in all twins, we believe the data included in our study was precise and accurate. Moreover, there are national guidelines for growth assessment that are widely spread and followed in our country [27], and each pair of twins was assessed by the same healthcare professional who is experienced and certified in the follow-up of these populations. Due to the overpowered sample size, trivial effects may have become statistically significant. Nevertheless, confidence intervals in most measurements are small and the results are consistent with published studies [1, 9, 10, 33]. Another limitation is that our study is based on hospital records and does not account for potential confounders such as parental height, socioeconomic factors, and dietary intake. We attempted to circumvent this limitation by analyzing maternal preconception body mass index in both monochorionic and dichorionic twins and found no significant difference. In addition, due to the absence of suitable growth charts for twins, we relied on singleton growth charts and singleton *z*-score scales. This may significantly impact the generalizability as the use of growth standards developed for singleton populations may not accurately reflect the unique growth trajectories of twins, particularly in the neonatal and early childhood periods. Nevertheless, this is an unavoidable limitation, and since twin pairs were measured in the same way, all growth changes and differences between the co-twins were preserved. Finally, although we analyzed head circumference z-scores, no neurodevelopmental outcomes were assessed. This omission is important, as neurodevelopmental outcomes could provide valuable insights into the long-term impact of growth discordance in twins. This may be an area of interest for future research.

In conclusion, inter-twin weight discordance was observed throughout the growth patterns of both larger and smaller twins, with the smaller discordant twins exhibiting adequate catch-up growth, resulting in no differences in any growth parameters compared to their larger co-twins by 36 months of CA. This study highlights the significant role of chorionicity in determining the timing of the resolution of growth disparities between twin pairs: monochorionic twins experienced persistent differences in weight, height, and head circumference until 18, 12, and 6 months of CA, respectively; in contrast, dichorionic twins showed resolution at later stages, with disparities lasting until 24, 18, and 12 months of CA, respectively. These results may reassure parents of discordant twins and healthcare professionals involved in the care of these co-twins, as smaller twins can be expected to reach their growth potential and catch up with their larger co-twins.

Further prospective and multicentric studies with standardized definitions are crucial to expanding our understanding of the impact of inter-twin weight discordance on the growth patterns among twin pairs with a special focus on neurodevelopmental outcomes.

## Data Availability

No datasets were generated or analysed during the current study.

## Abbreviations

ACOG: American College of Obstetricians and Gynecologists
ART: Assisted reproductive technologies
BW: Birth weight
CA: Corrected age
EFW: Estimated fetal weight
FGR: Fetal growth restriction
ISUOG: International Society of Ultrasound in Obstetrics and Gynecology
IQR: Interquartile range
NICU: Neonatal intensive care unit
SD: Standard deviation
TTTS: Twin-to-twin transfusion syndrome
WHO: World Health Organization
